# Association of Chewing Difficulty and Number of Remaining Teeth with Anxiety (GAD-7) Among Korean Adults: Evidence from the 2023 KNHANES

**DOI:** 10.3390/healthcare13212729

**Published:** 2025-10-28

**Authors:** Jun-Ha Kim, So-Yeong Kim

**Affiliations:** 1 Department of Oral & Maxillofacial Surgery, Graduate School, Kyung Hee University, Seoul 02447, Republic of Korea; kimjunhaa@khu.ac.kr; 2 Department of Preventive Medicine, College of Medicine, Chosun University, Gwangju 61452, Republic of Korea

**Keywords:** anxiety, GAD-7, oral health, number of remaining teeth, chewing difficulty, Korea National Health and Nutrition Examination Survey (KNHANES)

## Abstract

**Background**: Oral health is increasingly recognized as a determinant of overall well-being, but its role in mental health remains underexplored. Chewing difficulty and tooth loss can impair nutrition, social interaction, and quality of life, thereby contributing to psychological distress. **Objectives**: This study examined the association between oral health indicators and anxiety among Korean adults. **Methods**: Data were obtained from 4746 adults aged ≥19 years who participated in the 2023 Korea National Health and Nutrition Examination Survey (KNHANES). Anxiety was assessed using the Generalized Anxiety Disorder-7 (GAD-7), a validated 7-item self-report questionnaire with responses on a 4-point Likert scale (0 = not at all to 3 = nearly every day). Anxiety severity was categorized into four levels. Severity was categorized into four levels using the GAD-7. Oral health predictors included the number of remaining teeth and self-reported chewing difficulty, along with toothache experience, toothbrushing frequency, and unmet dental care needs. Complex survey-weighted ordinal logistic regression models were applied, adjusting for sociodemographic, behavioral, and clinical covariates. **Results**: Overall, 15.3% of adults reported mild, 3.1% moderate, and 1.6% severe anxiety. Chewing difficulty, fewer than 20 remaining teeth, overweight status, high stress, depressive symptoms, and unmet dental care needs were significantly associated with greater anxiety severity. **Conclusions**: The number of remaining teeth retention and chewing function were closely related to anxiety. Preserving functional dentition and ensuring timely access to dental care may be effective public health measures to reduce the psychological burden in the general population.

## 1. Introduction

Anxiety is one of the most common mental health conditions worldwide, affecting over 10% of the global population and leading to rising social and economic burdens [1]. In Korea, the prevalence of anxiety and depression has increased since the COVID-19 pandemic, compounded by population aging and socioeconomic disparities, making mental health an urgent public health challenge [2]. Oral health, beyond essential functions such as mastication and esthetics, plays a critical role in overall well-being. Poor oral conditions—including tooth loss, pain, and chewing difficulty—can restrict dietary intake, cause nutritional imbalance, and contribute to social withdrawal, thereby exacerbating psychological distress [3]. Locker reported that tooth loss and diminished oral function were significantly associated with poorer psychological well-being among older adults [4]. Globally, oral–mental health connections have been consistently documented. A systematic review and meta-analysis found that individuals with anxiety or depression had higher risks of periodontal disease and tooth loss compared with the general population, underscoring the concept of “no mental health without oral health” [5]. Despite this evidence, integrated strategies remain limited. In Korea, existing studies have primarily focused on depression or stress in relation to oral health. For example, Koh et al. reported that adults with fewer remaining teeth had higher levels of depression using data from the Korea National Health and Nutrition Examination Survey (KNHANES) [6].

This study hypothesized that oral health indicators, particularly chewing difficulty and the number of remaining teeth, would be significantly associated with higher levels of anxiety among Korean adults. Furthermore, we expected that inadequate dentition and impaired masticatory function may exacerbate psychological distress, even after accounting for sociodemographic and health-related covariates such as body weight. Recent evidence further supports this connection. For example, Hao et al. reported that tooth loss was associated with depression mediated by lifestyle and inflammation in a large cross-sectional study in China [7]. These findings underscore the importance of considering both physical and psychological consequences of oral health problems.

The number of remaining teeth and chewing ability are particularly important oral health indicators because they directly affect nutritional intake, physical health, and psychosocial well-being. Difficulty in chewing has been associated with dietary restrictions, reduced protein and fiber intake, and lower quality of life in older adults [8]. In addition, tooth loss is not only a functional impairment but also a visible condition that may lead to reduced self-esteem and social withdrawal [9]. These pathways suggest that number of remaining teeth and chewing difficulty may contribute to heightened psychological distress, including anxiety.

However, large-scale investigations on anxiety, particularly those using validated measures such as the Generalized Anxiety Disorder-7 (GAD-7), remain scarce. Against this background, the present study used nationally representative data from the 2023 KNHANES to examine the association between oral health indicators—including number of remaining teeth, chewing difficulty, toothache, oral hygiene behaviors, and unmet dental care needs—and anxiety. This study aimed to provide evidence for integrating oral health promotion into national mental health strategies.

## 2. Materials and Methods

This investigation was conducted as a cross-sectional study and was reported in compliance with the STROBE (Strengthening the Reporting of Observational Studies in Epidemiology) guidelines, which are intended to enhance transparency and quality in observational research [10].

### 2.1. Data Source and Study Population

We used data from the 2023 Korea National Health and Nutrition Examination Survey (KNHANES), an annual, nationally representative survey of the non-institutionalized Korean population conducted by the Korea Disease Control and Prevention Agency (KDCA). KNHANES applies a stratified, multistage, clustered probability sampling method. Health interviews are conducted by trained staff or via self-administered questionnaires, while health examinations are performed by medical professionals in mobile examination centers. Nutrition surveys are conducted using standardized protocols. All participants provide written informed consent, and each survey cycle is approved by the KDCA Institutional Review Board. For this study, we used the publicly available, de-identified 2023 dataset. The authors were not involved in data collection, and the secondary analysis was exempt from additional IRB review, in accordance with local regulations. Further details, including response rates and field procedures for the 2023 cycle, are available in the official KNHANES documentation published by KDCA [11].

We included adults aged ≥19 years who completed the health interview (including the GAD-7), underwent the oral health examination, and had non-missing values on primary exposures, outcome, and covariates. Participants with missing data for these key variables were excluded from the analytic sample.

### 2.2. Dependent Variable

The primary outcome was anxiety, measured with the Generalized Anxiety Disorder-7 (GAD-7) scale developed by Spitzer et al. [12]. The GAD-7 consists of seven items rated on a four-point scale (0 = not at all to 3 = nearly every day), with total scores ranging from 0 to 21. Higher scores indicate greater anxiety severity. Following the standard classification, scores were categorized into four groups: 0–4 = normal, 5–9 = mild, 10–14 = moderate, and 15–21 = severe anxiety. In KNHANES 2023 [11], the GAD-7 was administered via self-reported questionnaires, with trained staff available for clarification when necessary.

### 2.3. Independent Variables

The main predictors of interest in this study were chewing difficulty and the number of remaining teeth. Chewing discomfort was assessed through a self-administered question that asked participants whether they had ever experienced problems when chewing food. The number of natural teeth was categorized following the WHO’s definition of functional dentition, which considers 20 or more teeth as sufficient for proper mastication [13]. Based on this criterion, participants were grouped into four categories: edentulous (0 teeth), 1–19 teeth, 20–27 teeth, and 28 teeth (excluding third molars). This classification has been widely used in previous oral health research among older adults to distinguish between functional dentition and complete dentition [14,15].

In addition to these primary indicators, several supplementary oral health variables were included. The toothache experience was determined by asking whether the participant had suffered from tooth pain within the past year. Toothbrushing frequency was measured on the reported number of brushing occasions on the previous day and reclassified into two categories (<2 times and ≥2 times per day), based on prior literature [16]. Unmet dental care needs were evaluated with the question, “In the past year, did you require dental treatment but fail to receive it?”, and responses were categorized as no, yes, or dental care not required.

### 2.4. Covariates

Variables included sex, age (<65 vs. ≥65 years), residential area (urban/rural), educational attainment (elementary or less, middle/high school, ≥college), marital status (married vs. unmarried), household income (low, middle, high), and private health insurance status (yes/no). Age was dichotomized into <65 and ≥65 years in accordance with the Korean demographic transition and previous KNHANES-based epidemiological studies on oral and mental health. Although age could also be analyzed as a continuous variable, stratification at 65 years is widely applied to distinguish working-age from older populations.

Health-related covariates included physician-diagnosed hypertension and diabetes, smoking status (current smoker vs. non-smoker), and alcohol consumption (current drinker vs. non-drinker). According to the KNHANES protocol, former smokers and former drinkers were categorized as non-smokers and non-drinkers, respectively. Additional covariates included average sleep duration (adequate ≥ 7 h vs. insufficient < 7 h, based on WHO/CDC guidelines [17]), perceived stress (low vs. high), and depressive symptoms experienced within the past two weeks.

Physical activity was defined using the WHO 2020 guidelines [18]: ≥150 min/week of moderate-intensity, ≥75 min/week of vigorous activity, or an equivalent combination. Body mass index (BMI) was calculated as weight/height^2^ (kg/m^2^) and categorized per the WHO Asia-Pacific criteria: underweight (<18.5), normal (18.5–22.9), overweight (23.0–24.9), obese class I (25.0–29.9), and obese class II (≥30.0) [19].

### 2.5. Statistical Analysis

All analyses accounted for the complex survey design of KNHANES, including sampling weights, stratification, and clustering. Descriptive statistics were calculated as weighted proportions or means with standard errors. Group differences were tested using the Rao–Scott χ^2^ test for categorical variables and complex-sample t-tests or ANOVA for continuous variables. Ordinal logistic regression was performed to assess the association between anxiety severity (four GAD-7 categories) and oral health predictors, using the *svyolr* function in the R survey package. Three models were estimated: Model 1 (oral health indicators only), Model 2 (additionally adjusted for sociodemographic variables), and Model 3 (fully adjusted for behavioral and clinical covariates). Statistical significance was set at *p* < 0.05. Analyses were conducted in R version 4.5.0 (R Foundation for Statistical Computing, Vienna, Austria).

## 3. Results

### 3.1. Distribution of General Characteristics by Anxiety Severity

A total of 4746 subjects were studied, including 2048 male and 2698 Female. Anxiety levels were classified as minimal, mild, moderate, and severe based on the GAD-7 scale. The distribution of anxiety levels by general characteristics is shown below (Table 1). Females had higher rates of mild (59.3% vs. 40.7%) and severe (69.9% vs. 30.1%) anxiety than males (*p* < 0.001). The 19–64 age group had a higher rate of mild anxiety compared to the ≥65 age group (88.4%) (*p* < 0.001). Lower educational attainment was generally associated with higher rates of moderate and severe anxiety, although the highest proportion of severe anxiety was observed in the high school group (43.1%) (*p* = 0.013). Married individuals showed a higher prevalence of severe anxiety compared with the unmarried group (53.7% vs. 46.3%, *p* < 0.001) (Table 1).

### 3.2. Distribution of Health-Related Characteristics by Anxiety Severity

The distribution of anxiety levels by health-related characteristics is presented in Table 2. Those with hypertension had a higher prevalence of high anxiety (*p* = 0.014), and smokers also had a higher prevalence of high anxiety than non-smokers (23.1% vs. 17.2%, *p* = 0.060), although this association was not statistically significant. The group with high perceived stress levels had significantly higher rates of moderate (85.5%) and high anxiety (83.5%) (*p* < 0.001), and high anxiety was also very high in the group reporting depression at 78.7% (*p* < 0.001). Furthermore, anxiety levels were higher in the underweight and obese groups based on body mass index (BMI), with moderate anxiety being the highest at 12.6% in the severely obese group (*p* = 0.003). Post hoc analyses revealed that individuals in the underweight and obese II groups had a significantly higher prevalence of moderate-to-severe anxiety compared with the normal BMI group, whereas the overweight group showed a lower prevalence of anxiety (all *p* < 0.05) (Table 2).

### 3.3. Distribution of Oral Health-Related Characteristics by Anxiety Severity

The distribution of anxiety levels by oral health-related characteristics is presented in Table 3. The fewer the number of remaining teeth, the higher the anxiety level. The edentulous group, which comprised 1.5% of the total subjects, had a relatively high rate of high anxiety (*p* = 0.031). The group with toothache experience showed a higher rate of high anxiety (29.7%) than the group without toothache experience (70.3%) (*p* = 0.001). The group with chewing difficulties also had a significantly higher rate of severe anxiety (23.7%) (*p* < 0.001). In contrast, oral examinations and recent dental visits were not significantly associated with anxiety levels. However, the group with unmet dental care needs showed higher levels of mild (31.2%) and moderate anxiety (37.9%) (*p* < 0.001), and the rate of high anxiety was higher even when the toothbrushing frequency was less than twice a day (12.5% vs. 87.5%, *p* = 0.008) (Table 3).

**Figure 1:**
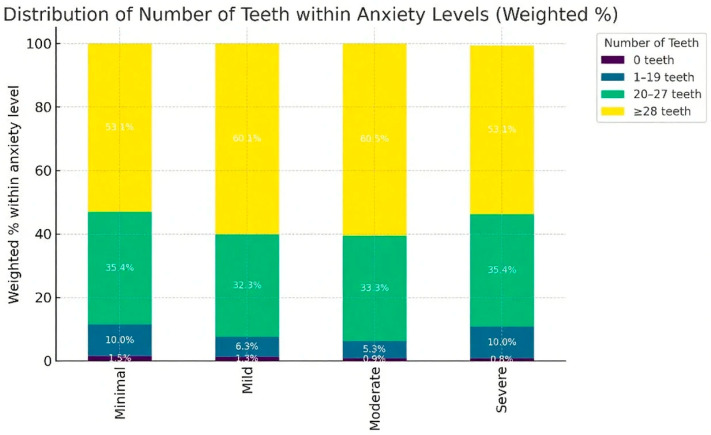
Weighted percentage distribution of anxiety severity (GAD-7 categories) according to number of remaining teeth among Korean adults aged ≥19 years, KNHANES 2023 [11]. *p*-values were obtained from the Rao–Scott χ^2^ test for group comparisons. Regression-based associations are additionally presented in Table 4.

As shown in Figure 1, the prevalence of higher anxiety severity was disproportionately greater among adults with fewer than 20 remaining teeth. In particular, the edentulous group demonstrated the highest proportion of moderate-to-severe anxiety. These graphical patterns further support the association between number of remaining teeth and psychological distress.

**Table 4:** Ordinal Logistic Regression of Anxiety (GAD-7 Categories).

Variable	Category	Model 1	*p*-Value	Model 2	*p*-Value	Model 3	*p*-Value
Number of teeth	≥28 (Ref)	1.00	–	1.00	–	1.00	–
	20–27	0.57 (0.30–1.06)	0.075	0.83 (0.42–1.63)	0.586	0.89 (0.70–1.12)	0.306
	1–19	0.47 (0.35–0.64)	<0.001	0.66 (0.47–0.93)	0.018	0.65 (0.43–0.99)	0.046
	0	0.77 (0.30–1.06)	0.075	0.83 (0.42–1.63)	0.586	0.54 (0.18–1.59)	0.263
Toothache	Yes (Ref)	1.00	–	1.00	–	1.00	–
	No	0.81 (0.67–0.99)	0.027	0.81 (0.67–0.98)	0.033	1.02 (0.82–1.26)	0.879
Chewing discomfort	Yes (Ref)	1.00	–	1.00	–	1.00	–
	No	0.55 (0.46–0.67)	<0.001	0.51 (0.41–0.61)	<0.001	0.79 (0.60–1.04)	0.087

OR = Odds Ratio, CI = Confidence Interval. Model 1: adjusted for oral health indicators. Model 2: additionally adjusted for sociodemographic factors. Model 3: fully adjusted for health behaviors and clinical variables.

### 3.4. Ordinal Logistic Regression of Anxiety (GAD-7 Categories)

The results of ordinal logistic regression analysis (Table 4) showed that in Model 1, the number of teeth, dental pain experience, and chewing discomfort were significantly associated with anxiety level. In particular, the anxiety level was significantly higher in patients with 1–19 remaining teeth compared to those with ≥28 teeth (OR = 0.47, 95% CI: 0.35–0.64, *p* < 0.001). In Model 2, even after adjusting for sociodemographic factors, the number of teeth (1–19, OR = 0.66, *p* = 0.018), gender (male, OR = 0.56, *p* < 0.001), age (<65 years, OR = 1.42, *p* = 0.004), and marital status (single, OR = 0.57, *p* < 0.001) remained significantly associated with anxiety level. In the final model (Model 3), the effects of number of teeth and chewing discomfort were reduced, whereas perceived stress level (OR = 0.18, *p* < 0.001), depressive experience (OR = 8.07, *p* < 0.001), overweight BMI (OR = 1.43, *p* = 0.031), and unmet dental experience (OR = 1.26, *p* = 0.009) were strongly associated with anxiety level (Table 4).

**Figure 2:**
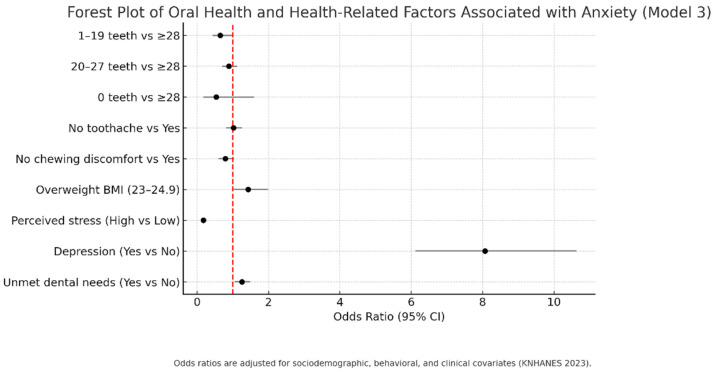
Adjusted odds ratios (Model 3) with 95% confidence intervals for the association between oral health indicators and anxiety severity (GAD-7 categories) among Korean adults, KNHANES 2023 [11]. Reference categories: ≥28 teeth, toothache = yes, chewing discomfort = yes. Forest Plot of Oral Health and Health-Related Factors Associated with Anxiety (Model 3). Each horizontal line represents the 95% confidence interval for the odds ratio, and the red dashed line indicates the reference value (OR = 1). Odds ratios are adjusted for sociodemographic, behavioral, and clinical covariates (KNHANES 2023).

The adjusted regression estimates are summarized in Figure 2. Adults with 1–19 remaining teeth had significantly higher odds of anxiety compared with those with ≥28 teeth, even after full adjustment. Chewing difficulty was also positively associated with anxiety, although its effect was attenuated in the fully adjusted model.

## 4. Discussion

This study examined the factors associated with anxiety among Korean adults, focusing on oral health status, health-related behaviors, and sociodemographic characteristics. The findings revealed that individuals with fewer remaining teeth, as well as those reporting chewing difficulties or dental pain, exhibited significantly higher levels of anxiety. Anxiety was also more prevalent among women, unmarried participants, and those experiencing high stress or depression. In addition, being overweight and reporting unmet dental care needs were both linked to elevated anxiety levels.

The graphical findings (Figure 1 and Figure 2) highlight that both the distribution of anxiety by number of remaining teeth and the adjusted associations in regression models converge on the same conclusion: functional dentition and chewing ability are important for mental health. These visualizations also underscore the disproportionate psychological burden carried by individuals with unmet dental care needs, suggesting that interventions aimed at preserving chewing function and reducing barriers to dental care may be associated with lower levels of anxiety reduction at the population level.

The association between the number of remaining teeth and anxiety aligns with earlier evidence. Having an adequate number of teeth influences not only mastication but also social functioning and self-esteem [20]. Tooth loss, conversely, may contribute to greater psychological distress and heightened anxiety [21]. In this study, participants with fewer than 20 teeth had elevated anxiety levels, consistent with the WHO benchmark for functional dentition (≥20 teeth) [9]. Notably, although the edentulous group represented a small proportion of the sample, they demonstrated disproportionately high anxiety levels, underscoring the broader psychological consequences of tooth loss.

Chewing impairment and oral pain also showed strong associations with anxiety. Beyond preventive and behavioral approaches, oral rehabilitation through prosthetic treatment and dental implants should also be considered. Restoring masticatory function via prosthetic interventions may alleviate anxiety symptoms by improving chewing ability, nutrition, and social interaction, which are closely linked to psychological well-being. Previous research indicates that oral discomfort can reduce daily functioning, restrict social interactions, and lead to nutritional imbalances, all of which may contribute to anxiety and depression [22,23]. The regression models in this study similarly confirmed that participants with chewing difficulties were more likely to report higher anxiety, reinforcing the notion that oral dysfunction may be an important factor to consider in mental health evaluation and care.

Stress perception and depressive symptoms demonstrated particularly strong relationships with anxiety. More than 80% of participants reporting high stress were categorized into moderate or severe anxiety groups, and nearly 79% of those with depression also experienced high anxiety. These results are in line with prior studies describing anxiety and depression as closely interrelated conditions within the same psychopathological spectrum [24]. The observation that depressive symptoms increased the odds of anxiety by more than eight-fold underscores the potential value of integrated clinical and public health approaches for addressing both disorders.

Anxiety was also more frequent among individuals classified as overweight (BMI 23.0–24.9), suggesting a potential U-shaped association, in which anxiety may be elevated in overweight groups relative to both normal and obese categories. This pattern may be influenced by body image concerns and experiences of social stigma [25]. Similarly, reporting unmet dental treatment needs was associated with higher anxiety levels, implying that barriers to accessing dental services or financial burdens may exacerbate psychological distress.

Interestingly, neither dental checkups nor recent dental visits showed significant associations with anxiety. This finding suggests that compliance with actual treatment may exert a stronger influence on mental health outcomes than routine participation in examinations alone.

The present study makes a meaningful contribution by comprehensively analyzing the relationship between oral health and anxiety using large-scale, nationally representative KNHANES data. The results emphasize that tooth loss, chewing problems, and unmet dental care needs are not only oral health concerns but also important for mental health. From a policy perspective, oral health initiatives may benefit from focusing on preserving dentition, and improving masticatory function in older adults, expanding dental service access through affordable preventive services, more community-based clinics, and strengthening the dental workforce may help reduce the psychological burden. Furthermore, dental practitioners should adopt a multidisciplinary approach that considers both physical and psychological aspects of patient care.

Several limitations must be acknowledged. First, the cross-sectional design prevents causal inferences between oral health and anxiety. Second, certain variables, including stress, depression, and sleep, were assessed through self-reported questionnaires, which may introduce reporting bias, and stress, depression, and sleep quality were assessed using single-item questions included in KNHANES, which may not fully capture these constructs. Future research should incorporate validated questionnaires, such as the Depression, Anxiety, and Stress Scale (DASS-21) for stress and depression and the Pittsburgh Sleep Quality Index (PSQI) for sleep, to improve measurement accuracy. Third, detailed dental characteristics, such as tooth position or prosthetic use, were not captured in this dataset. Despite these limitations, the use of representative national survey data provides valuable insights into the complex interactions between oral health, psychological well-being, and lifestyle factors, and offers important implications for clinical practice and public health policy. In this study, depressive symptoms were included as a covariate; however, it is possible that depression may also act as a mediator in the relationship between oral health and anxiety. This distinction could not be clearly determined in our cross-sectional design and should be addressed in future longitudinal studies.

Cultural perceptions of oral health and anxiety may shape both self-reporting and treatment-seeking behaviors. In East Asian contexts, sociocultural norms could influence oral health practices as well as the expression of anxiety, which should be considered when interpreting the findings.

## 5. Conclusions

This study provides evidence that both number of remaining teeth and chewing function are closely associated with anxiety among Korean adults. From a clinical and public health perspective, maintaining functional dentition and addressing masticatory difficulties may be associated with lower psychological burden. The findings suggest that oral health promotion has implications that extend beyond the prevention of dental disease, which may have implications for supporting mental health.

Integrating oral health management into mental healthcare frameworks may represent a comprehensive approach to improving overall well-being. Such integration emphasizes the importance of coordinated strategies that not only safeguard oral function but also may be linked to lower levels of anxiety in the population.

## Figures and Tables

**Table 1:** Distribution of General Characteristics by Anxiety Severity (GAD-7 Categories).

Variable	Category	Unweighted N	Minimal	Mild	Moderate	Severe	*p*-Value
Total		4746	72.5 (1.0)	18.6 (0.7)	6.3 (0.5)	2.6 (0.3)	-
Sex	Male	2048	51.2 (0.7)	40.7 (2.5)	38.6 (4.9)	30.1 (5.7)	<0.001
	Female	2698	48.8 (0.7)	59.3 (2.5)	61.4 (4.9)	69.9 (5.7)	
Age	19–64	3393	79.6 (0.9)	88.4 (1.4)	80.8 (3.2)	81.7 (3.9)	<0.001
	≥65	1353	20.4 (0.9)	11.6 (1.4)	19.2 (3.2)	18.3 (3.9)	
Residence	Urban	3806	83.7 (2.6)	84.6 (3.1)	83.0 (4.6)	88.9 (4.4)	0.674
	Rural	940	16.3 (2.6)	15.4 (3.1)	17.0 (4.6)	11.1 (4.4)	
Education	<Elementary	701	10.7 (0.7)	7.0 (1.1)	13.9 (2.7)	15.9 (4.7)	0.013
	Middle school	473	8.0 (0.5)	6.5 (1.2)	4.1 (1.7)	4.1 (2.2)	
	High school	1592	35.0 (1.1)	35.0 (2.5)	28.4 (4.5)	43.1 (6.4)	
	≥College	1980	46.3 (1.6)	51.5 (2.7)	53.6 (5.1)	36.9 (5.6)	
Marital status	Married	3842	75.5 (0.9)	63.8 (2.8)	70.6 (5.1)	53.7 (6.6)	<0.001
	Unmarried	904	24.5 (0.9)	36.2 (2.8)	29.4 (5.1)	46.3 (6.6)	
Household income	High	1485	25.4 (1.2)	27.2 (2.3)	31.4 (5.1)	40.0 (5.5)	0.190
	Middle	1010	22.0 (1.1)	22.2 (2.3)	19.9 (3.7)	14.4 (4.5)	
	Low	2251	52.5 (1.6)	50.5 (3.1)	48.6 (5.4)	45.5 (5.7)	
Private insurance	Yes	4028	87.7 (0.8)	90.4 (1.5)	80.1 (4.4)	81.3 (4.0)	0.011
	No	718	12.3 (0.8)	9.6 (1.5)	19.9 (4.4)	18.7 (4.0)	

Values are presented as weighted % (standard error, SE). Unmarried includes single, divorced, and widowed. *p*-values were obtained using Rao–Scott χ^2^ test for complex survey data.

**Table 2:** Distribution of Health-Related Characteristics by Anxiety Severity (GAD-7 Categories).

Variable	Category	Unweighted N	Minimal	Mild	Moderate	Severe	*p*-Value
Hypertension	No	3455	77.1 (0.9)	83.5 (1.7)	79.7 (3.6)	75.8 (5.6)	0.014
	Yes	1291	22.9 (0.9)	16.5 (1.7)	20.3 (3.6)	24.2 (5.6)	
Diabetes	No	4214	90.5 (0.6)	90.8 (1.3)	94.9 (1.7)	91.9 (2.8)	0.360
	Yes	532	9.5 (0.6)	9.2 (1.3)	5.1 (1.7)	8.1 (2.8)	
Smoking	Non-smoker	4020	82.8 (0.7)	77.2 (2.3)	82.0 (4.6)	76.9 (5.6)	0.060
	Smoker	726	17.2 (0.7)	22.8 (2.3)	18.0 (4.6)	23.1 (5.6)	
Alcohol use	Non-drinker	2282	44.3 (1.1)	43.4 (2.8)	53.8 (5.1)	43.3 (6.1)	0.314
	Drinker	2464	55.7 (1.1)	56.6 (2.8)	46.2 (5.1)	56.7 (6.1)	
Sleep duration	<7 h	2919	58.6 (1.0)	63.6 (2.3)	63.7 (5.0)	61.6 (5.6)	0.164
	≥7 h	1827	41.4 (1.0)	36.4 (2.3)	36.3 (5.0)	38.4 (5.6)	
Perceived stress	Low	3607	82.1 (0.7)	41.6 (2.6)	14.5 (2.9)	16.5 (4.4)	<0.001
	High	1139	17.9 (0.7)	58.4 (2.6)	85.5 (2.9)	83.5 (4.4)	
Depression	Yes	513	4.8 (0.4)	35.0 (2.5)	54.1 (5.7)	78.7 (4.7)	<0.001
	No	4233	95.2 (0.4)	65.0 (2.5)	45.9 (5.7)	21.3 (4.7)	
Physical activity	Inactive	2559	50.6 (1.1)	47.6 (2.5)	51.2 (5.1)	63.4 (5.7)	0.147
	Active	2187	49.4 (1.1)	52.4 (2.5)	48.8 (5.1)	36.6 (5.7)	
BMI	Underweight	219	4.1 (0.3)	7.4 (1.1)	8.5 (2.5)	4.9 (3.3)	0.003
	Normal	1750	35.2 (0.9)	40.3 (2.0)	37.8 (5.0)	43.7 (6.2)	
	Overweight	1040	22.4 (0.7)	18.6 (1.9)	14.7 (3.4)	18.0 (4.4)	
	Obese I	1419	30.8 (0.8)	25.4 (2.2)	26.4 (4.9)	24.9 (5.3)	
	Obese II	318	7.5 (0.6)	8.3 (1.4)	12.6 (3.1)	8.5 (3.6)	

Values are presented as weighted % (standard error, SE). *p*-values were obtained using Rao–Scott χ^2^ test for complex survey data.

**Table 3:** Distribution of Oral Health Characteristics by Anxiety Severity (GAD-7 Categories).

Variable	Category	Unweighted N	Minimal	Mild	Moderate	Severe	*p*-Value
Number of teeth	0	92	1.5 (0.2)	1.3 (0.4)	0.9 (0.5)	0.8 (0.8)	0.031
	1–19	599	10.0 (0.6)	6.3 (1.0)	5.3 (1.8)	10.0 (0.6)	
	20–27	1794	35.4 (0.9)	32.3 (2.1)	33.3 (4.7)	35.4 (0.9)	
	≥28	2261	53.1 (1.0)	60.1 (2.2)	60.5 (4.8)	53.1 (1.0)	
Toothache	No	3413	71.6 (1.4)	62.4 (2.9)	74.4 (4.1)	70.3 (5.9)	0.001
	Yes	1333	28.4 (1.4)	37.6 (2.9)	25.6 (4.1)	29.7 (5.9)	
Chewing discomfort	No	3983	86.7 (0.7)	81.0 (1.7)	80.3 (3.7)	76.3 (4.6)	<0.001
	Yes	763	13.3 (0.7)	19.0 (1.7)	19.7 (3.7)	23.7 (4.6)	
Dental check-up	No	2692	56.5 (1.0)	58.6 (2.6)	60.4 (5.6)	53.5 (6.7)	0.727
	Yes	2054	43.5 (1.0)	41.4 (2.6)	39.6 (5.6)	46.5 (6.7)	
Dental use	No	1910	40.8 (0.9)	41.9 (2.7)	41.3 (4.9)	41.7 (6.5)	0.981
	Yes	2836	59.2 (0.9)	58.1 (2.7)	58.7 (4.9)	58.3 (6.5)	
Unmet dental needs	Yes	1077	20.7 (0.8)	31.2 (2.2)	37.9 (4.7)	28.4 (5.7)	<0.001
	No	2501	53.6 (0.9)	47.7 (2.5)	46.7 (5.2)	52.6 (6.5)	
	Not needed	1168	25.7 (0.8)	21.1 (2.2)	15.3 (3.3)	19.0 (4.4)	
Toothbrushing frequency	<2/day	361	7.4 (0.5)	11.8 (1.8)	9.2 (2.7)	12.5 (3.8)	0.008
	≥2/day	4385	92.6 (0.5)	88.2 (1.8)	90.8 (2.7)	87.5 (3.8)	

Values are presented as weighted % (standard error, SE). *p*-values were obtained using Rao–Scott χ^2^ test for complex survey data.

## Data Availability

The data presented in this study are openly available in the Korea National Health and Nutrition Examination Survey (KNHANES) database at https://knhanes.kdca.go.kr, reference number: KNHANES 2023.
